# Parenting for Lifelong Health for Young Children in Montenegro: Preliminary Outcomes, Dissemination, and Broader Embedding of the Program

**DOI:** 10.1007/s11121-024-01682-x

**Published:** 2024-05-17

**Authors:** Judy Hutchings, Ida Ferdinandi, Roselinde Janowski, Catherine L. Ward, Amalee McCoy, Jamie Lachman, Frances Gardner, Margiad Elen Williams

**Affiliations:** 1 Present Address: Centre for Evidence-Based Early Intervention, Bangor University, Bangor, UK; 2 Present Address: UNICEF Country Office in Montenegro, Podgorica, Montenegro; 3 Present Address: Department of Psychology, and Safety and Violence Initiative, University of Cape Town, Cape Town, South Africa; 4 Present Address: Department of Social Policy and Intervention, University of Oxford, Oxford, UK; 5 Present Address: Peace Culture Foundation, Ban Waen, Thailand

**Keywords:** Implementation, Parent training, Child maltreatment, Prevention

## Abstract

The quality of parenting program implementation significantly affects the extent to which a program is delivered effectively as well as the likelihood of it becoming embedded in everyday services. The group based Parenting for Lifelong Health for Young Children (PLH-YC) program for parents of children aged 2–9 years was developed specifically for implementation in low- and middle-income contexts, has been tested in five randomized trials, and incorporates a number of strategies to encourage fidelity of delivery. This paper reports on the introduction of PLH-YC to Montenegro, including initial work to engage government agencies and service providers, adapt the program and, following initial evidence of effectiveness, implement strategies to promote effective delivery and embed the program. Following program adaptation and initial facilitator training, eight groups were run, supported with resources and supervision and independently evaluated. The successful pilot led to program training accreditation by national professional agencies and a series of steps to successfully further embed it into routine settings in Montenegro, including by recognizing the program in national policy documents. This led to further facilitator trainings, now numbering 97 facilitators and the certification of ten coaches and two trainers. By the end of 2023, 1278 parents, across 13 municipalities (half of all municipalities in Montenegro) and a range of service providers, have received the program. The paper describes the project phases and key fidelity components that underpinned the successful introduction and embedding of the program in Montenegro. The plan has resulted in Montenegro having its own domestic resources to continue to implement the program effectively and further plan for widespread dissemination.

Violence against children is a widespread phenomenon, with global prevalence of past year violence estimated as at least 50%, amounting to over 1 billion of the world’s children (Hillis et al., 2016). The consequences of such violence are both immediate and long-term, including aggression and anti-social behavior, lower cognitive ability, adverse mental health in both childhood and adulthood, criminal activity, and victimization and perpetration of intimate partner violence (Fulu et al., 2017). Parenting programs, based on social learning theory principles, prevent or reduce violence against children (Vlahovicova et al., 2017) with well-resourced early intervention trials in high-income countries (HICs) demonstrating reductions in both coercive and negative parenting and child conduct problems (Leijten et al., 2020). The need for such programs in low- and middle-income countries (LMICs) is even greater since levels of violence against children are higher (Hillis et al., 2016), and evidence is now emerging demonstrating that similar results can be achieved in these countries (Pedersen et al., 2019).

Unfortunately, even in HICs, there has been a failure to implement programs effectively at scale (Richter & Naicker, 2013) with interventions delivered in ongoing services by regular service staff typically achieving poorer results than those achieved in research trials (Gottfredson et al., 2015). Program developers may fail to include the necessary information for effective implementation by others; service providers may fail to deliver the entire program, “adapt” it in ways that reduce its effectiveness (Gottfredson et al., 2015), or lack the resources, suitably skilled or trained staff, and/or sufficient time for delivery (Gottfredson et al., 2006). This has led to the emergence of the field of implementation science (Britto et al., 2018; Flay et al., 2005; Mihalic et al., 2002), which explores the key essential components needed to effectively deliver evidence-based interventions and to subsequently embed them within services. Proctor et al. (2011) identified eight indicators related to the implementation of new programs, practices, and services: acceptability, adoption, appropriateness, feasibility, fidelity, implementation cost, penetration (or reach), and sustainability. A recent scoping review (Pinto et al., 2023) of the implementation of parenting programs in real-world settings found large variabilities in the reporting of different implementation indicators with some indicators reported frequently (e.g., fidelity and acceptability) and others rarely (e.g., cost). Knowing how programs are implemented in new and different contexts can help practitioners in selecting programs to deliver, improving services and outcomes for children and families. By addressing issues related to implementation (Berkel et al., 2011), recent studies of parenting programs (at least in HICs) show that delivery in routine practice can be achieved successfully and in some cases at scale (Hutchings, 2015).

## Parenting for Lifelong Health

Violence against children is more prevalent in LMICs as are the conditions that increase parenting challenges, including poverty, community violence, illness, early death, and related stressors (Parra-Cardona et al., 2021). Providing parent and caregiver support is key, as recognized by UNICEF (2014) and WHO (2016) strategies for protecting children from violence, and in turn is essential to the achievement of several of the United Nations Sustainable Development Goals (i.e., Goal 4 on promoting early childhood development, inclusive and equitable education and lifelong learning; Goal 5 on the empowerment of women and girls; and Goal 16 on promoting peaceful and inclusive societies).

While programs from HICs can be transported or transferred across countries to different cultures and contexts (Gardner et al., 2016), they are often too expensive to be delivered at scale in LMICs (Lansford et al., 2022; Mikton, 2012). In 2012, with support from the WHO and UNICEF, the Parenting for Lifelong Health (PLH) initiative was founded to develop and evaluate evidence informed interventions that also addressed issues of cost, transportability, and sustainability—or the continued use of programs in usual practice (Chambers et al., 2013; Ward et al., 2014). Since its inception, the initiative has focused on developing and disseminating a suite of culturally adaptable, freely available, and rigorously tested parenting programs designed for low-resource settings to enhance parenting, promote child development, and prevent violence against children (Clarke, 2019).

The PLH program for parents of Young Children aged 2–9 (PLH-YC) was initially developed by the first and sixth authors and colleagues with, and for, socially disadvantaged families in South Africa. Four main principles guided its development:

(i) Incorporation of effective, evidence-based core components grounded in social learning principles: The well-established effective content components include child-led play, praise and rewards, limit setting, and non-violent strategies to manage problem behavior (Leijten et al., 2019).
(ii) Collaborative delivery style using core social learning theory delivery principles (Eames et al., 2010; Hutchings et al., 2004): The program is delivered collaboratively and focuses on parent empowerment by addressing parents’ own goals through group discussion, rehearsal of skills through role play, and home assignments and includes materials to support a collaborative facilitator style.
(iii) The need to ensure that the program was low cost and adaptable for delivery to local cultures and contexts (Lachman et al., 2016): The program is licensed with a Creative Commons Attribution, Non-Commercial, No Derivatives license, and detailed manuals are available to download via the WHO website (https://www.who.int/teams/social-determinants-of-health/parenting-for-lifelong-health). Materials are low-cost and easily adaptable to new settings (e.g., cartoon strips showing positive and problematic parenting behaviors rather than video vignettes).
(iv) Tools for ensuring effective delivery and replication (Hutchings et al., 2007b): The program includes facilitator training and certification, ongoing consultation and supervision, and materials for service providers, facilitators, and parents that are cornerstones of effectively taking a program to scale (Gottfredson et al., 2015). Attendance registers record parental engagement and are a valuable tool for service providers to demonstrate whether parents are retained, a key measure of whether what they receive aligns with their expectations (Goldiamond, 1975). Checklists monitor both content and process components of delivery and are explored in supervision, where facilitators present their challenges and goals and review video footage of group sessions. An implementation guide for service providers supports fidelity, while skills checklists are used by trainers to assess facilitators for certification (WHO, 2020).

PLH-YC is a group-based parenting program delivered by two trained co-facilitators to groups of up to 15 parents, with each session lasting 2–2.5 h. The program uses the metaphor of building a “House of Support,” in which the walls represent positive parenting and the roof represents limit setting and discipline strategies. Weekly sessions cover spending quality time with children, naming feelings and actions, using praise and rewards, giving instructions, establishing household rules, non-violent discipline techniques, and problem-solving. The final session reviews what parents have learned, focuses on how to continue applying learned skills at home, and ends with a celebration. Facilitators make mid-week phone calls to parents to monitor their use of skills at home and engagement. The program was initially tested in a large randomized controlled trial (RCT) in South Africa that found that intervention parents used more positive parenting and their children were more likely to behave positively towards their parents (Ward et al., 2020). The program has since been adapted and used in other LMICs across Africa, Asia, and Eastern Europe, where it has been tested in an additional four RCTs (Lachman et al., 2017; Taut et al., 2021), including a trial in the Philippines using the 12 session program (Lachman et al., 2021) and an eight session version in Thailand (McCoy et al., 2021).

## The Context in Montenegro

Montenegro is an upper-middle-income country, with a population of around 630,000. It was granted European Union candidacy status in 2010 and has embarked on an intensive series of reforms, with a key priority in “*the rule of law and fundamental rights sector*” (Hamilton et al., 2018). Following amendments to the Family Law prohibiting the violent punishment of children, including by parents and caregivers, in 2017 UNICEF and Ministers from the Montenegrin Government co-hosted a conference “End Violence against Children.” The conference was attended by the President of Montenegro. On that occasion, the first national Strategy for the Prevention and Protection of Children from Violence (2017–2021) and a national campaign to protect children from adverse childhood experiences were launched. The campaign aimed to (1) break the taboo around adverse childhood experiences and family violence, (2) increase awareness of the harm of violence on child development both in the short- and long-term and of its broader impact on society, and (3) call for professionals and parents to unite in raising children without violence. This was the first time that parenting interventions were debated at a national level in Montenegro.

## Introducing the Program in Montenegro

Given the need for more studies describing the implementation of programs in new settings (Pinto et al., 2023), this paper reports on the introduction, set-up and subsequent steps to embed the PLH-YC program into existing service delivery systems in Montenegro. The plan was set up in line with UNICEF’s subsequently published program implementation framework that identifies a nine-step process for program implementation (UNICEF, 2021). This framework is similar to other published implementation frameworks (see synthesis by Meyers et al., 2012). The steps include the following: (1) conduct need assessment; (2) identify the program’s target population(s); (3) build coalitions that will join in advocacy for an enabling environment; (4) agree on delivery platforms; (5) identify the “parenting workforce”; (6) enhance demand generation; (7) pilot, adapt, and implement; (8) ensure monitoring and evaluation; and (9) develop detailed plans for taking parenting programs to scale.

### Step 1: The Need—Prevalence of the Problem

Violent discipline (physical punishment and psychological aggression) is widespread in Montenegro and has been experienced by 66% of children aged 1 to 14 (MONSTAT & UNICEF, 2019). While 31% of children aged 1 to 14 experience physical punishment, only 10% of mothers/caretakers think that physical punishment is necessary (ibid). This may imply that high levels of physical punishment are mostly due to a lack of skills among parents for positive parenting.

### Step 2: Identify the Target Population

Given the known levels of violent discipline and the broad government initiative to address this, the decision was taken to initially offer the program through any interested existing service provider agency and to parents on a universal basis.

### Step 3: Coalition Building

With financial assistance from the European Union, UNICEF has acted as the necessary local champion in Montenegro, providing the key initiative in publicizing the program and initiating discussions with the relevant ministries and service providers across a range of health, education and NGO service settings. UNICEF already had strong working relationships with both government agencies and service providers through years of cooperation agreements with the Government of Montenegro. It also engaged service providers as partners in the implementation plan from the outset throughout the project and kept government agencies informed.

### Steps 4 and 5: Agreeing on Delivery and Identifying the Program Delivery Workforce

To ensure that delivery was feasible, UNICEF explained the program requirements and support available in stakeholder meetings prior to partners committing to the project. UNICEF, with financial support of the European Union, committed to fund adaptation and translation of manuals, the provision of training, supervision of program facilitators, and an independent pre-post evaluation of the first set of groups that included the independent collection of post-intervention data. UNICEF also committed to provide a small grant to agencies to support initial implementation, for instance for the purchase of cameras to film sessions for presentation at facilitator supervision. Participating agencies agreed to cover staff time to attend training, recruit parents, collect baseline measures, deliver the program and attend supervision, and provide workshop materials and refreshments for parents.

The ways in which UNICEF supported the components of implementation involved recruitment (step 6), piloting (step 7), and monitoring and evaluation (step 8). Subsequent plans for scale-up of the program involved the development of an implementation strategy (step 9).

## Implementation

**Fig. 1:**
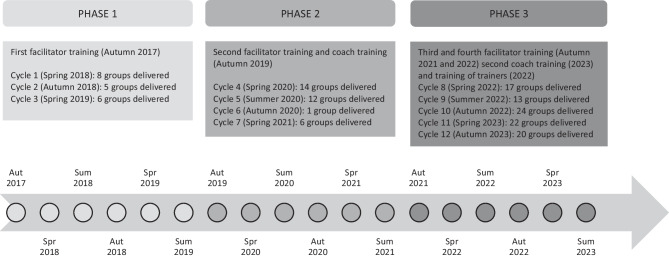
Timeline of delivery phases and cycles

The implementation of the PLH-YC program adopted by UNICEF in Montenegro was divided into three phases described below. Initial components of the implementation involved procedures for the recruitment and set-up with services followed by initial delivery (phase 1). Later phases of implementation involved strategies for sustaining/embedding the program in Montenegro (phases 2 and 3). A timeline of the three phases and embedded cycles is shown in Fig. 1. As part of routine implementation of PLH-YC programs, facilitators completed satisfaction forms at the end of training, collected information on recruitment, enrolment, attendance and completion for the groups, demographic information about group participants, and parental satisfaction at the end of group delivery. This information was stored by UNICEF and shared with the researchers.

### Phase 1: Preparation and Initial Implementation

Once sufficient agency interest had been generated, the project was confirmed and the preparation phase began. This included adapting the program to the Montenegrin context. Surface level adaptation primarily involved redrawing the illustrated stories for a European audience and renaming the characters, although the scripts of the stories remained largely unchanged. The translation was checked in the UK by a Montenegrin psychology lecturer at Bangor University.

UNICEF’s goal, from the outset, assuming initial evidence of program effectiveness in Montenegro, was to develop a cadre of program facilitators from whom, with delivery experience, coaches and trainers would be identified. This would allow Montenegro to have in-country resources to train and support additional facilitators and be able to embed the program and take it to scale.

In Autumn 2017, the initial four-day training was delivered in Montenegro to 24 program facilitators. Training and supervision were delivered in English by the first and sixth authors with the support of an interpreter provided by UNICEF. A workshop for program facilitators and their service managers was held at the end of the training to confirm the resources needed for effective program implementation and to ensure that managers understood the program content, delivery structure, resources, and time commitment needed for facilitators to deliver the program effectively.

The 24 professionals who attended facilitator training included 12 psychologists, six educators, five nurses, and a social worker from 11 agencies (see Table 1). Agencies included five primary healthcare centers, three NGOs, two kindergartens, and one child behavior center. Evaluations of the training were very positive with 85% of the responses to the 13 evaluation items indicating that trainees were “very” or “completely” satisfied.

**Table 1:** Program facilitators

Training	# trained	# new agencies	# new municipalities	Profession			
				Psychologist	Educator^b^	Social worker	Nurse
1st training (phase 1)	24	11	4	12	6	1	5
2nd training (phase 2)	24	7	1	6	10	3	5
3rd training (phase 3)	26	7	2	6	13	3^c^	3
4th training (phase 3)	24	5	6	4	15	3	2
Total	97^a^	30	13	28	44	9	15

^a^One facilitator attended the training twice (once in phase 1 and then phase 3) due to a long break

^b^Includes special educator, preschool teacher, and speech therapist

^c^Includes one political scientist

Following training, facilitators delivered three cycles of groups (see Table 2). In cycle 1, 17 facilitators (71% of those trained) delivered eight groups in their respective settings in Spring 2018. In cycle 2 (Autumn 2018), 12 facilitators (83% of whom had delivered in cycle 1) delivered five groups, and in cycle 3 (Spring 2019), 13 facilitators delivered six groups (77% of whom had delivered in both previous cycles and 23% had delivered in either cycle 1 or cycle 2). These included staff in primary healthcare centers, NGOs, and a kindergarten setting in four Montenegrin municipalities.

**Table 2:** Levels of parent engagement in phases 1 to 3

**Cycles**	***N*** **groups**	***N*** **recruited**	***n*** **enrolled**^**a**^	***n*** **completed**^**b**^ (%)^**c**^
**Phase 1**				
1	8	82	75	65 (86.7)
2	5	55	52	49 (94.2)
3	6	64	61	53 (86.9)
Total phase 1	19	201	188	167 (88.8)
**Phase 2**				
4	14	145	142	125 (88.0)
5	12	124	121	112 (92.6)
6	1	7	7	6 (85.7)
7	6	69	66	62 (93.9)
Total phase 2	33	345	336	305 (90.8)
**Phase 3**				
8	17	171	159	135 (84.9)
9	13	136	128	119 (93.0)
10	24	249	235	195 (83.0)
11	22	231	225	195 (86.7)
12	19	203	185	162 (87.6)
Total phase 3	95	990	932	806 (86.5)

^a^Attend at least 1 session

^b^Attend at least 7 sessions

^c^Based on enrolled participants

Step 6 of the implementation framework relates to recruitment. The target population for the groups was varied. Given the diverse backgrounds and responsibilities of the facilitators and the lack of inclusion criteria, one fifth (19%) of the parents reported children with clinical levels of behavioral problems. Recruitment strategies differed depending on the setting. Primary healthcare settings used websites, word of mouth, leaflets in waiting rooms and corridors, and approached parents accessing services. Education settings used social media and websites as well as approached parents in need of more support. NGOs used their existing networks to reach out to vulnerable families and those already accessing support groups, as well as social media and direct communication. There were also some cross-setting referrals such as between healthcare and social work settings and between NGOs and kindergartens.

As cycle 1 was the first time any of the facilitators had delivered the intervention, the supervision arrangements were intensive. All group sessions were videotaped, and eight supervision sessions were delivered to facilitators by the first author during initial program delivery: three days in-person and five days remotely via videoconferencing. Facilitators prepared material for supervision, including weekly fidelity checklists, and identified a challenge and a goal for the upcoming supervision session and presented a brief section of the videotaped parenting group session that depicted the challenge. Solutions were brainstormed and roleplayed. Challenges brought to supervision during the earlier part of the program mainly included process issues, such as helping parents to set clear goals, managing talkative parents, ensuring the inclusion of quiet parents, and engaging parents in roleplay activities. Later challenges mainly focused on the latter part of program content, such as addressing the management of challenging behavior. Over the eight days of supervision, each facilitator had the opportunity to present a challenge and receive supervision on four occasions. At least one facilitator from each group was required to attend supervision but, in all but one case, both facilitators from each group attended all the sessions (95% attendance).

Groups were delivered weekly over 12 sessions. Program engagement was very high with 65 (87%) of the 75 enrolled parents completing the program (see Table 2). Completion was defined as having attended at least seven of the 12 sessions. The first delivery of the program (cycle 1) was used to pilot the intervention (steps 7 and 8 of the implementation framework) with outcomes collected from families. Results from this pilot are reported elsewhere (McCoy, 2021).

In December 2018, following a national UNICEF conference in July 2018 at which the outcomes from the successful pilot were presented, the Committee for Human Rights and Freedoms of the Parliament of Montenegro recommended scaling up the program to all municipalities in the country (Report from the 27th session of the Committee for Human Rights and Freedoms, Number 00–63-8/18–41/ from 25 December 2018). Additionally, the facilitator training was accredited by the National Institute for Social and Child Protection (Decision on accreditation 03–25/2, 19 November 2018, renewed in 2023 Decision 03–128/23–9/2). In the meantime, based on both facilitator enthusiasm for the program and feedback of positive initial outcome data, the participating agencies agreed that two further cycles of groups would be delivered. Program engagement for both cycles remained high (see Table 2). After consultation with the involved agencies and relevant ministries, UNICEF decided to continue supporting the program to enable further expansion and develop a pool of national coaches and trainers.

### Phase 2: The Second Facilitator Training and Development of a Pool of Coaches

Phases 2 and 3 of the project represent step 9 of the implementation framework (planning for scale-up involving monitoring and evaluation and planning). In September 2019, a second cohort of 24 facilitators was trained by the first author, including facilitators from seven new agencies across five municipalities (see Table 1). In this phase, four further cycles of groups were delivered between Autumn 2019 and Spring 2021, reaching 336 parents. Some of these cycles of program delivery were affected by the COVID-19 pandemic restrictions, with half of the sessions in both Spring 2020 and Autumn 2020 delivered online due to lockdown. Despite these challenges, program engagement remained high at 91% completion rates (see Table 2).

In October 2019, six phase 1 facilitators, who had each delivered at least three groups, were trained by the first author as coaches to support the new cohort of facilitators. The coaches worked in pairs to provide supervision to new facilitators. At the same time, they received a mixture of live and remote supervision based on a review of videos of their coaching sessions. Evaluations of the coach training were very positive with all responses to the eight items rated as “very” or “completely” helpful. In 2020, the six coaches, who had by then each supervised facilitators through delivery of two groups of the program, all attained certification as coaches based on detailed feedback rated from subtitled videotapes of a full coaching session, demonstrating high levels of fidelity.

### Phase 3: Third and Fourth Facilitator Trainings, Expanding the Pool of Coaches, and Development of Trainers

In 2021, due to COVID-19 restrictions, training of the third group of 26 program facilitators was provided using a “hybrid” (both in-person and online) format, with training delivered remotely by the first author. The trainee facilitators were at one site and supported by two certified coaches who were identified to be trained as trainers. The remainder of phase 3 took place later in 2022 and in 2023. This included training the fourth group of 24 facilitators by the trainee trainers, under live supervision from the first author, after which they were certified to deliver future trainings as well as to deliver training to six new coaches to support the growing number of facilitators. Five coaches were certified in spring 2023. By the end of Phase 3, the program had been delivered to a further 932 parents of whom 806 (86.5%) had completed the program (see Table 2).

### Current Situation and National Achievements

By Autumn 2023, 97 facilitators have been trained from 30 different agencies in 12 municipalities across Montenegro, and 1278 parents have received certificates of program completion.

In 2019, the program was accredited by the Bureau for Education (Decision of the National Council for Education 023–1161/2019–31 from 17 July 2019), and the accreditation was subsequently renewed (Decision of the National Council for Education 01–011/22–819/3 from 19 September 2022). In 2020, in addition to the earlier facilitator training accreditation, the National Institute for Social and Child Protection accredited the coach training (Decision on accreditation 03–4/1 from 6 May 2020, amended by the Decision on accreditation from 03–128/23–13/2). Further success was achieved when the Ministry of Education Strategy for Early and Preschool Education 2021–2025 included the expansion of the PLH-YC program from eight (in 2021) to 12 pre-school service providers by 2025. Furthermore, the first national Early Childhood Development Strategy, adopted by the government in October 2023, envisages program promotion and expansion, as well as program adaptation to reach specific target groups.

### Implementation Indicators

Table 3 displays the operational definitions for the implementation indicators used for the project. For adoption, 97 facilitators have been trained in the program across a total of 30 agencies in 12 municipalities in Montenegro. This represents half of all municipalities in the country. In terms of the feasibility, parent engagement (number of parents completing the program) has remained consistently above 80% throughout all cycles in all three phases with a range of 83% to 94%. For reach, 50% of trained facilitators in phase 1 were psychologists with 25% as educators, 21% nurses, and 4% social workers. In phase 2, there is a shift to more educators (42%) which is unsurprising given the accreditation of the program by the Bureau of Education in July 2019. The number of social workers also increased to 12.5% which again coincides with the accreditation of the facilitator training by the National Institute of Social and Child Protection (November 2018). In phase 3, the number of educators increases further to 56% which coincides with the Ministry of Education Strategy for expanding into further pre-schools. In terms of families, over the three phases, 1526 parents completed pre-program demographic surveys administered by the facilitators. The average age of parents was 35 years, and 11.3% were fathers. More than half had a child aged 2–9 years who was a boy (58%). Almost 1 in 5 (19%) of parents reported at least one of the following difficulties in their respective households: food insecurity (running out of money for food or essentials in the last month); adult illness (an adult who is very unwell—in hospital or in bed a lot of the time); child illness (a child in the household who is very unwell); potential child disability (a child who has trouble hearing, seeing, talking or walking, or who struggles at school); household conflict (problematic arguments with shouting or hitting); and alcohol.

**Table 3:** Operational definitions of implementation indicators

**Implementation indicator**	**Description**	**Operationalization**
Adoption	Intention or initial decision to implement the program	Numbers of facilitators completing training and agencies joining implementation
Feasibility	Extent to which program is successfully implemented	Parent completion of the program
Reach	Integration of the program within a service setting	Diversity in facilitators and agencies, numbers of families reached

## Discussion

The launch of the PLH program in Montenegro arose from a clear need as well as a policy orientation set by the Government of Montenegro to prohibit all forms of violence against children in line with international legal commitments. Years of successful cooperation with UNICEF and support by the European Union were instrumental to its materialization. Impressively, this is one of few implementations of a parenting program delivered in a routine setting for universal prevention of violence against children in an LMIC from which evidence of effective delivery of an evidence-based program has led to broader scaling up and government recognition.

The initial parenting groups were successfully delivered and participant satisfaction was high. Enrollment and completion rates were higher than those achieved in rigorous trials in South Africa (Lachman et al., 2017, 2018; Ward et al., 2020) and were consistent with rates reported from programs of similar length in HICs (Chacko et al., 2016).

Program retention is associated with the quality of implementation (Flay et al., 2005; Lansford et al., 2022; Mihalic et al., 2002) and whether programs meet parents’ expectations and needs (Goldiamond, 1975). The results on implementation, retention, and satisfaction rates all suggest that this was the case. This led to the recommendation by the Parliamentary Committee to scale up the program, plans for program expansion in government policy, accreditation by the relevant Child Protection and Education agencies, and further support for scaling up by UNICEF and the European Union.

There is promising evidence of sustainability of the program with many of the original facilitators having continued to implement the program. In the report commissioned by UNICEF (McCoy, 2021), facilitators reported that their ability to do this in a collaborative manner had been considerably enhanced by the supervision process, particularly while delivering the program for a second time when they were able to more fully understand and develop collaborative delivery skills. Given that group work with parents requires skilled facilitation to establish and maintain relationships, as some parents can be both challenged and challenging (Hutchings et al., 2004), ongoing coaching assisted facilitators in gaining experience and a more sophisticated grasp of the program components, particularly the collaborative delivery process (McCoy, 2021). This confirmed that with a focus on supervision and implementation fidelity, good program outcomes are similarly possible in routine service settings in LMICs as they are in HICs (Hutchings, 2015).

The impressive early results prompted UNICEF to invest in the subsequent phases, including boosting political will to take the program to scale (Shiffman & Smith, 2007). With 1278 parents having received certificates of completion by the end of 2023, as well as a sizeable cadre of professionals—97 facilitators trained and 10 coaches and two in-house trainers accredited—an important start has been made in embedding the program in Montenegro. This has supported the government commitment to incorporate the program into routine practices and policies on a wider scale (Loening-Voysey et al., 2018).

Several factors contributed to the successful implementation and subsequent embedding of the program in Montenegro:

1. UNICEF was the program champion in Montenegro (Hutchings et al., 2004; Shiffman & Smith, 2007; Taylor & Biglan, 1998), with a strong campaigning focus on preventing and reducing violence against children.
2. UNICEF has had a history of decades of successful cooperation with the Government of Montenegro and public institutions and civil society organizations in the area of child rights.
3. The Government—particularly the Ministry of Education, Science and Innovation, the Ministry of Labour and Social Welfare, and the Ministry of Health—has had a high level of commitment to protecting children from violence and to providing parenting support. An important context for government support across multiple ministries was their commitment to human rights, and candidature for European Union accession.
4. UNICEF acted as the liaison with the PLH implementation team. With assistance from the European Union, it funded program adaptation, facilitator training and supervision, and supported program delivery in a variety of ways, such as keeping track of implementation throughout the process, ensuring fidelity with a high level of video-based supervision, and promoting consistent facilitator record keeping.
5. The staff were professionally qualified and employed by agencies that could support program delivery. The evidence from the initial delivery of the program (cycle 1), as well as the enthusiasm of the facilitators, helped the agencies to recognize the potential benefits for their service users, and the high quality of both the program and supervision provided strong motivation for delivering it as a form of professional development.
6. Service managers were engaged as partners from the outset and were clear about what was required from them as implementing agencies, including resources and the time required to deliver the program as well as what was being provided by UNICEF.
7. Service providers demonstrated a strong personal commitment to the program and reported substantial benefits for their clients.
8. UNICEF recognized the importance of determining whether the intervention worked to deliver their goals of reducing violence against children through funding the evaluation and follow-up data collection from the initial eight groups (cycle 1) by an independent researcher.

The reasons for the impressive achievement can also be understood in terms of the need to adhere to five core fidelity principles (Mihalic et al., 2002):

1. The program was evidence-based for the target population (parents of 2–9-year-old children).
2. A range of strategies were used to recruit the target population based on the knowledge of the trained facilitators.
3. Service access to the program was facilitated by the Government of Montenegro.
4. UNICEF support ensured implementation fidelity through program adaptation, training, and supervision, while recruitment of agencies was supported by the Ministry of Health and the Ministry of Education.
5. The initial delivery of the program was evaluated in terms of both delivery and outcomes.

Stakeholder meetings before service providers’ commitment to the plan ensured that they were partners and understood their roles and expectations. Program delivery as part of routine services from the outset involved their recognition that achieving the program goals was part of their core business. This likely contributed to its integration and continued delivery (Loening-Voysey et al., 2018) once the initial outcomes were available. Presentation of the results at a conference in June 2018 helped to ensure that the initial achievements were effectively disseminated and led to recognition by the Montenegrin Parliament Committee for Human Rights and Freedoms. The accreditation of facilitator training made the program more appealing to professionals as a recognized means of continuing professional development.

The initial results contributed to the program becoming embedded, with trained facilitators showing enthusiasm and in turn being supported by their agencies for ongoing delivery. The role of the European Union and UNICEF in providing funding, but also requiring services to allocate staff time to deliver it and attend supervision, was essential. A study (Hutchings et al., 2007a) of a different parenting program similarly led to that program continuing to be embedded in agency service delivery over 15 years later. In comparison, a study (Little et al., 2012) that provided additional funding for facilitators to deliver the same parenting program failed to embed the program, with facilitators reverting to their old jobs at the end of the study when there was no longer funding to deliver the program, despite positive outcomes. This suggests that agency commitment of resources and allocation of time required for delivery from the outset may be an important factor in ensuring maintenance of effective programs once they receive feedback on program effectiveness (i.e., that it is also meeting their goals).

Some limitations need to be acknowledged. Although useful implementation indicators are reported, fidelity of program delivery was not directly measured due to lack of funding and staff time to do this. This may have impacted on how the program was delivered as program drift (unplanned deviations from the intervention protocol) is relatively common (Sanetti et al., 2021). However, facilitators received intensive supervision throughout all three phases from an experienced trainer (first author) and subsequently from trained in-house coaches and trainers which can help guard against intervention drift. A shortage of staff and staff time in the various agencies meant that the program was competing with other agency staff time. Continued advocacy for funding is necessary as scale-up is dependent on managers being willing to prioritize allocation of staff time to deliver the program over other aspects of their work, or in some cases to pay for staff time to deliver it. The positive outcomes and the staff enthusiasm for the program contributed to agency willingness to continue with the program. Program scaling-up is defined in the national ECD Strategy adopted in 2023. This strategy is costed, but program-based budgeting is developing slowly, so the available budget for delivery remains unclear.

## Conclusion

The preliminary effectiveness in routine service delivery settings in Montenegro led to the decision by the Government, UNICEF, and partners to continue the project. The recommendation by the Parliament of Montenegro for national scale-up of PLH-YC, as well as the inclusion of the program in government policy, suggests the potential of the program to achieve widespread dissemination, which would make Montenegro a global leader in the wide-scale prevention of violence against children. A strong cadre of trained and certified facilitators, coaches, and trainers has made Montenegro self-sufficient in their efforts to embed the program further, and accreditation of the training at various levels should further encourage services to provide support for staff to continue to offer the program to parents.
